# Echogenic perfluorohexane-loaded macrophages adhere *in vivo* to activated vascular endothelium in mice, an explorative study

**DOI:** 10.1186/1476-7120-13-1

**Published:** 2015-01-08

**Authors:** Liselotte M Kornmann, Alma Zernecke, Daniëlle MJ Curfs, Ben JA Janssen, Christian Weber, Menno PJ de Winther, Robert S Reneman, Arnold PG Hoeks, Koen D Reesink

**Affiliations:** Department of Biomedical Engineering, Cardiovascular Research Institute Maastricht, Maastricht University, PO Box 616, 6200 Maastricht, MD The Netherlands; Institute for Molecular Cardiovascular Research, University Hospital Aachen, RWTH Aachen University, Aachen, Germany; Department of Molecular Genetics, Cardiovascular Research Institute Maastricht, Maastricht University, Maastricht, The Netherlands; Department of Pharmacology, Cardiovascular Research Institute Maastricht, Maastricht University, Maastricht, The Netherlands; Department of Physiology, Cardiovascular Research Institute Maastricht, Maastricht University, Maastricht, The Netherlands

**Keywords:** Atherosclerosis, Ultrasound contrast, Macrophage, Endothelium, Perfluorocarbon

## Abstract

**Background:**

Macrophages may concentrate ultrasound contrast agents and exhibit selective adhesion to activated endothelium. The present study investigates in mice the potential of perfluorohexane (PFH) loaded macrophages to act as ultrasound contrast agent with high reflectivity and specifically targeted at (atherosclerotic) vascular lesions.

**Methods:**

Lung passage was evaluated with a mouse echo scanner after injection, at a slow pace or as a bolus, of varying doses of PFH-loaded and unloaded bone marrow macrophages (BMM) into the jugular vein. The interaction of PFH-loaded and unloaded BMM with TNF-α stimulated carotid artery endothelium after tail vein injection was assessed by means of intravital microscopy.

**Results:**

High doses of jugular vein injected PFH-loaded BMM were visible with ultrasound in the pulmonary artery and detectable in the carotid artery. At intravital microscopy, tail vein injected BMM exhibited rolling and adhesion behavior at the TNF-α stimulated carotid endothelium, similar to that of native blood leukocytes. Rolling behavior was not different between PFH-loaded and unloaded BMM (p = 0.38).

**Conclusion:**

*In vivo,* perfluorohexane loaded macrophages pass the pulmonary circulation and appear on the arterial side. Moreover, they roll and adhere selectively to activated endothelium under physiological flow conditions. These findings indicate that perfluorohexane loaded BMM could be used to study processes *in vivo* where endothelial activation plays a role, such as atherosclerosis.

## Background

Atherosclerosis is an inflammatory disease and is initiated by the activation and dysfunction of endothelial cells by mediators, such as hyperlipidemia and shear stress [1, 2]. Predilection sites for the development of atherosclerotic plaques are areas opposite to the flow divider in such branch points as the carotid artery bulb, where blood flow is disturbed, local wall shear stress is bidirectional and average wall shear stress is low [3]. Dysfunctional endothelial cells express pro-inflammatory adhesion molecules, which subsequently mediate initial attachment, restrained rolling and firm adhesion of monocytes and other leukocytes to the activated endothelium. Both initial attachment and rolling are predominantly regulated by selectins and their respective carbohydrate ligands [4]. Monocytes may detach and be released back into the blood stream, but may also invade the vessel wall, transform into macrophages and scavenge inflammatory, necrotic material and fat to become foam cells.

Early detection of athero-prone sites might help to identify people at risk for cardiovascular events like stroke and myocardial infarction. Ultrasound molecular imaging utilizes ultrasound contrast agents [5] that carry specific adhesion molecules (e.g. antibodies) on their surface, facilitating binding to such specific targets as atherogenic areas on the arterial wall [6–9]. In the past decade, experimental and clinical validation studies have shown that for the microcirculation targeted ultrasound contrast agents, such as echogenic liposomes, microbubbles and perfluorocarbon emulsions, do improve visualization of specific structures [10–13]. These results have led to high expectations for dedicated molecular ultrasound imaging of activated endothelium of large and middle-sized arteries. The optimistic view emanating from many of the studies on adhesion properties of ultrasound contrast agents [14–16], however, should be interpreted with care [17]. Studies were usually performed in flow chambers after static incubation or under unphysiologically low shear stress (0.02-0.5 Pa) conditions [7, 9, 18, 19]. Moreover, the contrast agents used exhibited low capture and weak adhesion efficiency even at low shear stresses. Especially in small rodents, the adherence requirements for ultrasound contrast agents are very demanding. In small animals, mean wall shear stress in the common carotid artery is about 8 Pa [20, 21], which is substantially higher than the 1.2 Pa reported for the human carotid artery [22, 23].

Considering the difficulties related to adhesion encountered with molecularly targeted contrast agents *in vivo*, circulatory cells like monocytes may serve as a potential alternative. Monocytes are naturally equipped to adhere selectively to activated endothelium and to resist, while adhering, physiological shear stresses in large- and middle-sized arteries. Studies with noninvasive techniques like PET/CT have demonstrated the feasibility of monocytes as a contrast agent vehicle for *in vivo* imaging [24, 25].

We previously reported on the *in vitro* potential of monocytes to act as a targeted vehicle for ultrasound contrast agents [26]. Using murine primary bone marrow derived macrophages (BMM), we demonstrated that the echogenicity of these cells is dose-dependently related to their perfluorohexane uptake while loaded BMM maintain their functional adhesive properties under static conditions.

Perfluorohexane emulsions have a specific mass of 1.7 kg/l, a boiling point of 56°C (http://www.rsc.org/learn-chemistry/wiki/Substance:Perfluorohexane), and a sound speed of 521 m/s at 37°C [27], and, therefore, an acoustic impedance of 0.875 MRayl. Considering the acoustic impedance mismatch between perfluorohexane in its fluid phase and blood or tissue (about 1.5 MRayl), perfluorohexane (PFH) exhibits substantial reflectivity *in vivo*, though lower than air filled contrast bubbles. The small particle size of PFH emulsions with an average diameter on the order of 0.3 μm [28, 29] further limits reflectivity, unless local site-specific surface accumulation is achieved [30]. In our approach, volume accumulation of PFH emulsions is achieved by phagocytosis where the conglomerate within a macrophage exhibits enhanced reflectivity. It should be noted, that PFH emulsions remain in the fluid state under normal ultrasound exposure [31, 32], and, hence, unlike microbubbles, do not suffer from gas diffusion, nor are they sensitive to (repetitive) ultrasound interrogation. Preparation of the emulsions one day ahead and a 3 hour loading process do not change the emulsions characteristics [26], while viscoelastic damping by the macrophage does not appear to alter ultrasound reflectivity [33]. Moreover, 24 hours after loading no significant changes in BMM adhesive properties and in emulsion echogenicity could be observed [26]. The relatively long lifespan may allow for a prolonged time for site-specific accumulation of PFH loaded macrophages to achieve local echo enhancement, superseding the reflectivity of surrounding blood and tissue.

As monocytes *in vitro* are stimulated to convert into macrophages and are allowed to phagocytose PFH emulsions, they may become larger and stiffer. Leukocytes and macrophages have a cell size considerably larger than the diameter of capillaries [34]. Despite the mismatch in size, those large white blood cells are capable to pass the microcirculation by deformation, although the passage time is larger than that of blood plasma or red blood cells [35]. In blood, leukocytes are abundantly available, showing similar adhesion behavior, and, hence, are the first choice to compare with (loaded) macrophages, especially because they are able to pass the lung continuously.

In the present series of experiments, we aim to obtain proof of principle for the applicability of PFH loaded BMM as an ultrasound contrast agent *in vivo. In vitro* we study whether loaded BMM are different in diameter compared to blood leukocytes. In mice studies we investigate *in vivo* whether PFH loaded BMM (1) are able to pass the pulmonary circulation, (2) interact with cytokine stimulated endothelial cells of the carotid artery to achieve selective accumulation *in vivo*, and (3) show selective adhesion to stimulated endothelium under realistic blood flow conditions.

## Materials and methods

### Preparation of PFH emulsions

The PFH emulsions were composed of 40% v/v PFH (C_6_F_14_; Sigma-Aldrich, Steinheim, Germany) and a surfactant co-mixture (0.5% w/v) as previously described [26]. The selected surfactant concentration governs the eventual size distribution of the emulsion, i.e. an average diameter of 0.3 μm and a similar distribution width [29]. To remove liposomes and non-incorporated lipids the samples were washed and centrifuged at 2000 g for 30 minutes. PFH emulsions were resuspended in 2 mL phosphate buffer saline (PBS) and kept under nitrogen at 4°C until use.

### Loading of BMM with PFH emulsions

BMM were obtained from C57/BL6 mice and cultured according to standard procedures [36, 37]. BMMs were seeded in 6-well plates (2×10^6^/well) and left overnight to adhere at 37°C in a 5% CO_2_ incubator. The next day, cells were incubated with 2% or 4% v/v PFH emulsions for 3 hours at 37°C in a 5% CO_2_ incubator. After PFH loading, BMM were washed to remove non-incorporated PFH emulsions. Next, cells were lifted using 4 mg/mL lidocaine dissolved in PBS-10 mM EDTA according to standard procedures [38]. Cells were centrifuged at 300 g for 5 min at 4°C, which removed any residual emulsions and cell debris as verified by FACS analysis (fluorescence-activated cell sorting). Cell viability was monitored microscopically and by counting the number of viable cells upon harvesting. The loaded BMM were kept on ice (4°C) until the experiment on the same day.

### Cell diameter distribution

Loaded (2% v/v PFH) and unloaded (0% v/v PFH) BMM were dispersed in phosphate buffer solution (PBS) and fixated in a solution of 3% paraformaldehyde (Merck VWR, Amsterdam, the Netherlands) for 15 minutes. Fixated BMM were centrifuged at 300 g for 5 min at 4°C, dissolved in PBS and added to a Petri dish well for diameter distribution measurements. After allowing the BMM to adhere to the bottom, Petri-dishes were diametrically scanned with a microscope (Nikon Eclipse E800, Japan) with a 20× objective in an upright position. Murine blood smears, stained with May-Grunwald solution and Giemsa solution, were used as reference in leukocyte typing (neutrophils, lymphocytes and monocytes) and in the determination of the diameter distributions. For each of the above conditions, 200 cells were evaluated and measured using Image Pro Plus (Media Cybernetics Inc., Silver Spring, MD, USA).

### Animals

Male and female C57/BL6 J mice (16 to 20-week old, 20 to 30 grams) were obtained from Charles River Laboratories (Maastricht, the Netherlands, and Sulzfeld Germany). Mice were fed a normal diet and were allowed to drink water ad libitum. The experiments were approved by the institutional animal care and use committee of Maastricht University, Maastricht, the Netherlands and the Landesamt für Natur, Umwelt und Verbraucherschutz Nordrhein-Westfalen, Germany. Mice were anesthetized by subcutaneous administration of a mixture of xylazine (15 mg Xylazin/kg body weight; Ceva Sante Animale, Naaldwijk, the Netherlands) and ketamine (75 mg Nimatek/kg body weight; Eurovet, Cuijk, the Netherlands).

### Echo enhancement in blood

Pilot studies were conducted to study BMM echo enhancement *in vivo*. For this purpose, 15 mice were anesthetized, the chest was shaved, and a venous line (PE10) was inserted into the right jugular vein. Through this catheter, doses of 2 to 15 million of unloaded or loaded (2% or 4% v/v) BMM, suspended in 150 μl RPMI-1640 with 2% fetal calf serum and 5 U/ml heparin, were administered manually either as a bolus injection (1 s) or by slow infusion (30 s). Ultrasound imaging was performed with an ultra-high frequency (30 MHz) mechanical sector imaging system (Vevo 770, Visual Sonics, Toronto, Ontario, Canada) with an axial resolution of 55 μm. The ultrasound system was operated in standard B-mode with the emission power setting at 100%. The probe, fixed in a mechanical arm, was placed on the chest (transmission gel applied) to provide either a simultaneous view of the aorta, pulmonary artery and left ventricle or a view of the carotid artery. Evaluation of the Vevo 770 brightness calibration bar revealed that the grey-scale presentation was on a linear scale. Echo gains of the system were held constant during imaging. Continuous B-mode video recording were made at a frame rate of 25 Hz, covering baseline, injection and distribution over the blood pool of loaded and unloaded BMM. Ultrasound movies were processed off-line using ImagePro software (Media Cybernetics, Silverspring, MD, USA) to extract the blood echo level for all frames at a single pixel situated in the pulmonary artery, aorta or carotid artery. In a final processing stage the intensity waveform was smoothed (window 10 seconds) and converted to dB with the average baseline level as reference.

### In vivo interactions of leukocytes and BMM with the endothelium

As described by others, in arteries leukocyte-endothelium interactions are virtually absent without cytokine stimulation [39]. The significance of TNF-α (tumor necrosis factor) stimulation for leukocyte interaction with the endothelium was verified by injecting intraperitoneally TNF-α (1 μg/mL; PeproTec,London, UK) in 6 mice and PBS in 6 mice (control) six hours before imaging. After induction of anesthesia, the left carotid artery was surgically exposed and the exposed tissues were kept moist with PBS throughout the experiment. Blood leukocytes were visualized *in vivo* by injecting Rhodamine 6G (1 μL, 0.02%; Molecular Probes, Karlsruhe, Germany) via the tail vein. After 20 minutes to allow binding of Rhodamine to leukocytes, interactions of leukocytes with the carotid artery endothelium were visualized *in situ* by means of a Zeiss Axiotech microscope (20× water-immersion objective, Carl Zeiss, Oberkochen, Germany) with a 100 W HBO mercury lamp (Osram, Eichstätt, Germany), using epi-illumination. Excitation wavelength for fluorescence imaging was 526 nm. Video images were obtained from the anterior wall of the common and the external carotid artery and at the level of the carotid bifurcation. At each location two video recordings of 5 seconds were acquired at a frame rate of 25 Hz and saved on hard disk.

In a subsequent series of experiments, we tested the adhesion properties of both loaded and unloaded BMM, labeled *ex-vivo* with Rhodamine 6G and washed three times with PBS. Four mice were stimulated six hours before imaging with intraperitoneal TNF-α. In 2 mice, 2.5 million loaded (2% v/v) and in another 2 mice unloaded BMM were injected manually into the tail vein as a bolus. The tail vein is more easily accessible and it provides a better dilution than the jugular vein, reducing the likelihood of lung congestion [35]. Two minutes after injection, interactions of the BMM with the carotid artery endothelium were visualized *in situ*. The observation and recording procedure was the same as for the native leukocytes (see above).

Video images of leukocytes and BMM interactions with the artery wall were evaluated off-line, using ImagePro software. The number of BMM rolling over or adhering to the endothelium was determined per recording by two independent observers (LK and KR). Rolling distance was defined as the distance a rolling cell covered within the 5-second duration of the video recording and within the field of view, before leaving the field of view or before detaching from the endothelium. Rolling velocity was defined as the rolling distance divided by the transit time (number of frames divided by the frame rate). Firm adhesion was defined as a cell remaining stationary during the 5-second recording time**.** Cells attaching or rolling only very briefly (<2 s) were not taken into account in the number of rolling or firmly adhering cells, neither were they included in the assessment of rolling distance and velocity, as they likely do not contribute to accumulation and, hence, enhancement of the echo signals at the site of action.

### Statistical analysis

Data are presented as median and range, unless stated otherwise. Differences in distribution variance were tested by F-test. Two-sample, two-sided Student t-tests, assuming unequal variances, were performed on the data to detect statistical differences in cell diameter distributions. Differences in BMM rolling and in adhesion behavior were evaluated with the Mann–Whitney U-test. Calculations were performed using GraphPad Prism (GraphPad Software Inc, San Diego, CA, USA) and Excel (Microsoft, Redmond, WA, USA) software. A p-value <0.05 was considered statistically significant.

## Results

### Cell diameter

**Figure 1:**
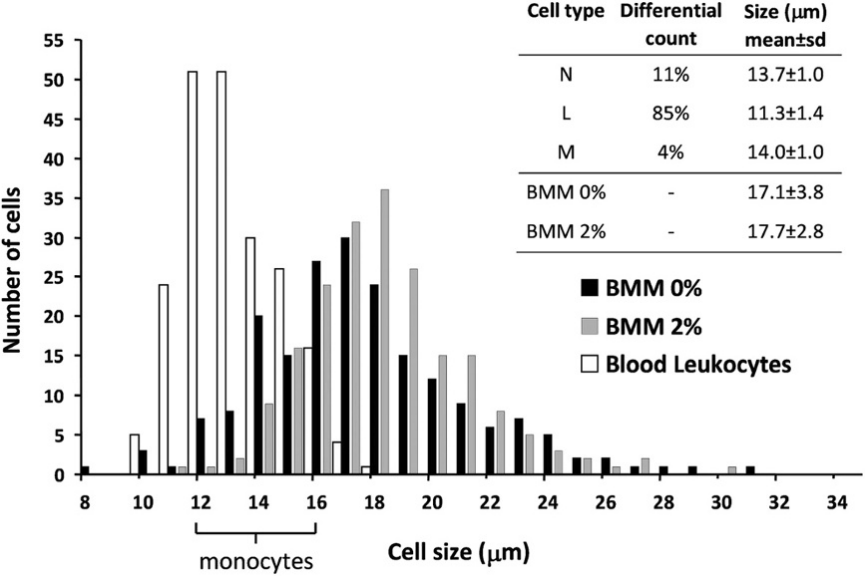
**Diameter distribution of mouse blood leukocytes (N, neutrophil; L, lymphocyte; M, monocyte) and cultured bone marrow macrophages (BMM).** Histograms show a broad diameter distribution for both PFH loaded (2%) and unloaded (0%) BMM compared to blood leukocytes. The bracket on the x-axis indicates the approximate diameter range of blood monocytes. n = 200 cells, for each group.

BMM diameter was significantly larger (p < 0.001) and more disperse (p < 0.001) than that of blood leukocytes (Figure 1). There was a borderline significant difference in average diameter (p = 0.049) between 2% PFH loaded (n = 200, mean ± SD = 17.7 ± 2.8 μm, median 17.4 μm) and unloaded BMM (n = 200, 17.1 ± 3.8 μm, median 16.6 μm), but the distribution width of the loaded cells was smaller than that of the unloaded ones (p < 0.001). Overall, both loaded and unloaded BMM were larger than blood leukocytes (n = 200, mean ± SD = 14.0 ± 1.0 μm), but the distributions were overlapping in the 12–16 μm diameter range.

### Echo enhancement in blood

Ultrasound B-mode images showed no blood echo enhancement in the pulmonary artery after injection of unloaded BMM into the jugular vein (n = 8).

**Figure 2:**
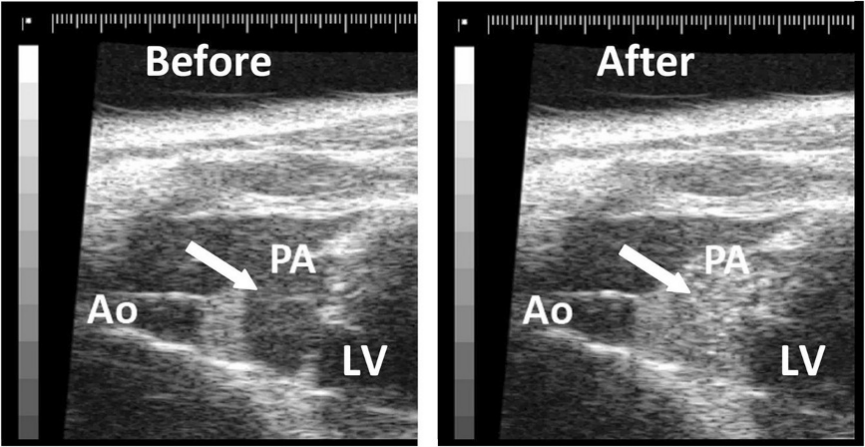
**Ultrasound images of a mouse heart before (left) and after (right) jugular vein injection of 7 million PFH (2% v/v) loaded BMM.** Right panel shows a clear enhancement (indicated with an arrow) in the pulmonary artery (PA) compared to the aorta (Ao) and the left ventricle (LV).

**Figure 3:**
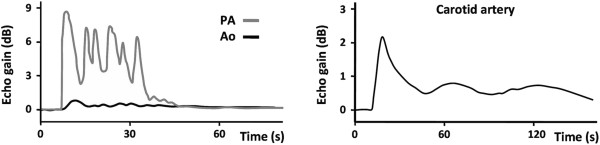
**Left: blood enhancement in the pulmonary artery (PA) and aorta (Ao) after a slow injection (30 s) of 15 million 4% v/v loaded BMM into the jugular vein.** Right: transient increase of blood echogenicity enhancement in the carotid artery just after a bolus injection of 15 million of PFH (4% v/v) loaded BMM into the jugular vein.

In 3 mice we injected 5 to 7 million 2% loaded BMM. Of these three mice, one got unintentionally a spurious BMM injection, while for the other the experiment was terminated because of problems with the anesthesia set-up. In the third mouse (7 million BMM, slow infusion) we observed a notable transient echo enhancement in the pulmonary artery (Figure 2).4 Mice got an injection of 4% loaded BMM with a dose ranging from 2–15 million cells. Slow infusion of 2 million loaded cells did not induce echo enhancement in the aorta (mouse 1), but 7 million loaded cells (slow infusion) caused an increase in echo level of 0.8 dB in the pulmonary artery and of 0.2 dB in the aorta (mouse 2). Slow injection of a high dose of 15 million 4% loaded BMM (mouse 3) temporarily enhanced the blood echogenicity in the pulmonary artery with 9 dB (Figure 3, left panel) and induced a small, but detectable enhancement in the aorta (1 dB peak). Also a bolus injection of a large number (15 million) of 4% loaded BMM (mouse 4) caused a substantial, but transient blood echogenicity enhancement of 2 dB in the common carotid artery of one mouse (Figure 3, right panel). The above results indicate that at least part of the injected BMM do pass the pulmonary circulation and arrive on the arterial side.

### In vivo interactions of leukocytes and BMM with the endothelium

As described by others [39], in arteries leukocyte-endothelium interactions are regulated by cytokine stimulation. Intravital microscopy of carotid arteries showed that in unstimulated mice (n = 6, peritoneal PBS injection), an average count of 1 adhering native leukocytes (median 1, range 0–5) was observed (36 recordings in total). TNF-α stimulation (n = 6; 36 recordings) resulted in adhesion of significantly (one-sided p = 0.01) more leukocytes (median 4, range 0–45) to the endothelium.

**Figure 4:**
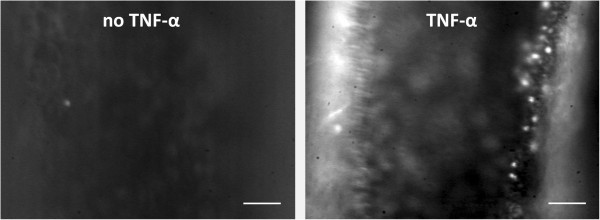
**Intravital fluorescence microscopy images of the mouse carotid artery.** Interactions of leukocytes are virtually absent in unstimulated mice (left). TNF-α stimulation effectively increases the number of leukocytes interacting with the endothelium (right). In the right picture the left wall of the artery is out of focus. Leukocytes are Rhodamine labeled. Scale bar = 50 μm.

Interactions of injected BMM with the TNF-α stimulated endothelium (totaling 36 loaded and 30 unloaded BMM) are illustrated in Figure 4. The trajectory could be identified over the image for 21 PFH loaded and 7 unloaded BMM. PFH loading had no significant effect on the rolling velocity or rolling distance of BMM (Table 1). Similarly, there was no difference in the incidence of stably adhering cells (8 loaded and 5 unloaded BMM). The number of cells attaching or rolling only very briefly during a recording was 11 for PFH loaded BMM and 13 for unloaded BMM.

**Table 1:** ***In vivo,*** **loaded and unloaded BMM behave similarly considering rolling velocity and rolling distance (reported as mean ± SD)**

	Unloaded BMM	Loaded BMM (2% v/v)	P
n	7	21	
Rolling velocity (μm/s)	33 ± 14	39 ± 14	0.18
Rolling distance (μm)	130 ± 55	114 ± 16	0.38

All **16** animals survived and were sacrificed after imaging, indicating that the applied dose (2.5 million for unloaded and 2% v/v loaded BMM) was tolerated by the animals.

## Discussion

The present pilot study was performed to investigate *in vivo* the potential of monocytes to act as ultrasound contrast agent. Intravenously administered perfluorohexane loaded bone marrow macrophages were able to pass the lung circulation in mice. Most importantly, we observed that loaded macrophages exhibited rolling and selective adhesion behavior at the TNF-α stimulated endothelium of the carotid artery wall, similar to unloaded macrophages.

The echogenicity of emulsions with a sub-micron diameter is limited; hence, those particles are unsuited to act as a blood pool contrast agent. Despite the size limitation, we were able to visualize loaded BMM (2% and 4% v/v) in the pulmonary artery, even though the background level is quite high (Figure 2). However, in the carotid arteries they were only visible after bolus injection of a high dose of BMM with maximal PFH loading (4% v/v). The enhanced echogenicity can be explained by the accumulation of echogenic material within the macrophage, resulting in a diameter increase of 0.6 μm corresponding to a volume increase of about 10%. As a consequence we are no longer dealing with sub-micron particles but with a conglomeration. A major advantage of loading macrophages with small particles is that the loaded macrophages retain deformability. This is essential for the passage through the microcirculation and for the adherence to the activated endothelium by maximizing the contact area and lowering the exposure to the prevailing shear stress.

Monocytes and other leukocytes are naturally equipped to roll on and selectively adhere to activated endothelium and to resist physiological shear stresses in large- and middle-sized arteries. Bonds formed between endothelial selectins and integrins on the one hand, and monocyte ligands on the other may result in rolling and eventually adhesion [40, 41]. These observations corroborate our findings that in the mouse carotid artery, native leukocyte adhesion is indeed enhanced by TNF-α stimulation. Similarly, both perfluorohexane loaded and unloaded bone marrow macrophages roll on and adhere to the artery wall through the interaction with selectins and integrins, which are expressed after TNF-α stimulation. In a previous study, we showed that PFH-loading of bone marrow macrophages did not affect the presence of PSGL-1, VLA-4, Mac-1 and LFA-1 on the cell surface, nor their ability to adhere to TNF-α stimulated endothelium under stationary (no shear) conditions [26]. The present study shows that in the *in vivo* situation perfluorohexane loaded and unloaded bone marrow macrophages also comply with this notion. Moreover, loading BMM with perfluorohexane did not degrade adhesion affinity as loaded BMM exhibited similar stable adhesion as unloaded BMM (8 and 5 BMM, respectively).

As reported, the PFH loaded BMM show only for extremely high doses, administered in a short time, a notable increase in blood pool echogenicity. Consequently, the enhanced echogenicity by local accumulation of PFH loaded BMM at stimulated sites will not be obscured by the echo level of the blood pool.

The low number of adhering and rolling BMM observed in the present study is likely a consequence of the protocol followed. To avoid ambiguity about the extent and homogeneity of local stimulation we used systemic application of TNF-α, which inherently enhances adhesion throughout the body and therefore reduces the number of BMM available for adhesion within the region of our interest. Moreover, the relatively large amount of native blood cells already adhering to the stimulated wall before the BMM are injected, occupy available adhesion sites. Moreover, in the current study the accumulation time was less than 10 minutes because of the acute nature of the experiments. Allowing prolonged exposure, which presents no problems for the stable emulsions, would surely contribute to accumulation.

A good alternative for macrophages derived from bone marrow might be isolated blood monocytes, because they are smaller than cultured macrophages, which lowers the chance of being captured in the lung and may decrease the sensitivity to shear stress. A limitation of using isolated monocytes, however, would be the number of donor mice needed to perform an ultrasound molecular imaging study in mouse models of atherosclerosis. Bone marrow isolation allows retrieval of a far higher number of BMM from a single mouse. In humans, ex-vivo radio-labeled leukocytes are in use clinically for scintigraphic imaging of inflammatory and infectious processes [24, 25], where leukocytes are obtained from the (same) patient.

## Conclusions

The current proof of principle study clearly demonstrates that, *in vivo,* PFH-loaded macrophages circulate and are able to roll and adhere selectively to stimulated carotid endothelium under physiological shear stress conditions. Therefore, we conclude that PFH-loaded monocytes may have a potential to be used as a targeted ultrasound contrast agent. Further investigations are required to gain more insight into the relationship between local expression of molecular markers on endothelial surfaces and the local concentration and diameter of adhered ultrasound contrast agents (i.e. loaded monocytes).

## Authors’ information

LK, AH & KR: Biomedical Engineering, Cardiovascular Research Institute Maastricht, Maastricht University, Maastricht, the Netherlands.

DC & MdW: Molecular Genetics, Cardiovascular Research Institute Maastricht, Maastricht University, Maastricht, the Netherlands.

BJ: Pharmacology, Cardiovascular Research Institute Maastricht, Maastricht University, Maastricht, the Netherlands.

RR: Physiology. Cardiovascular Research Institute Maastricht, Maastricht University, Maastricht, the Netherlands.

AZ & CW: Institute for Molecular Cardiovascular Research, University Hospital Aachen, RWTH Aachen University, Aachen, Germany.

## Abbreviations

BMM: Bone marrow derived macrophages
B-mode: Brightness mode
FACS: Fluorescence-activated cell sorting
Mac-1: Macrophage-1 antigen
LFA-1: Lymphocyte function-associated antigen 1
PBS: Phosphate buffer saline
PFH: Perfluorohexane (C_6_F_14_)
PSGL-1: P-Selectin glycoprotein ligand-1
Rayl: Unit of acoustic impedance (Pa.s/m)
TNF: Tumor necrosis factor
VLA-4: Very late antigen-4.
